# Additive Effects of 3,4-Methylenedioxymethamphetamine (MDMA) and Compassionate Imagery on Self-Compassion in Recreational Users of Ecstasy

**DOI:** 10.1007/s12671-017-0849-0

**Published:** 2017-11-04

**Authors:** Sunjeev K. Kamboj, Ylva S. E. Walldén, Caroline J. Falconer, Majdah Raji Alotaibi, Ian S. Blagbrough, Stephen M. Husbands, Tom P. Freeman

**Affiliations:** 1 Clinical Psychopharmacology Unit, Research Department of Clinical, Educational and Health Psychology, University College London, Gower St., London, WC1E 6BT UK; 2 Medicinal Chemistry, Department of Pharmacy and Pharmacology, University of Bath, Bath, BA2 7AY UK; 3 Present Address: National Addiction Centre, Institute of Psychiatry, Psychology and Neuroscience, King’s College London, 4 Windsor Walk, London, SE5 8BB UK

**Keywords:** MDMA, Prosocial, Compassion, Self-compassion, Self-criticism, Compassionate imagery, Empathy

## Abstract

3,4-Methylenedioxymethylamphetamine (MDMA;‘ecstasy’) produces prosocial subjective effects that may extend to affiliative feelings towards *the self*. Behavioural techniques can produce similar self-directed affiliation. For example, compassionate imagery (CI) and ecstasy reduce self-criticism and increase self-compassion to a similar extent, with the effects of CI enhanced in the presence of ecstasy. Here, we examine self-compassion and self-criticism in recreational users who consumed chemically verified MDMA in a within-subjects crossover study. In a naturalistic setting, polydrug-using participants performed a self-focused CI exercise on two occasions separated by ≥6 days: once having consumed self-sourced MDMA and once not. Effects on state self-criticism, self-compassion and emotional empathy were assessed before and after MDMA use (or over an extended baseline period on the occasion that MDMA was not consumed) and reassessed after CI. In participants (*n* = 20; 8 women) whose ecstasy contained MDMA and no other drug, CI and MDMA appeared to separately increase emotional empathy (to critical facial expressions) and self-compassion. The effects of CI and MDMA on self-compassion also appeared to be additive. Establishing the observed effects in controlled studies will be critical for determining the combined utility of these approaches in fostering adaptive self-attitudes in a therapeutic context.

## Introduction

Ecstasy is a popular recreational drug (European Monitoring Centre for Drugs and Drug Addiction 2015). Its subjective interpersonal effects are well documented in recreational users (Sumnall et al. 2006), with reports of heightened interpersonal understanding and compassion for others. Similar subjective effects are seen in controlled laboratory experiments with 3,4-methylenedioxymethamphetamine (MDMA; Kamilar-Britt and Bedi 2015), the primary constituent of most street ecstasy (Brunt et al. 2012).

MDMA may have unique potential as an adjunct to psychotherapy for disorders characterised by heightened (interpersonal) threat, concerns about social evaluation and deficits in understanding of interpersonal communication (Danforth et al. 2016; Mithoefer et al. 2016). A number of biological and neuropsychological mechanisms have been proposed to account for these potential therapeutic effects. Behavioural studies, for example, indicate that MDMA increases emotional empathy and reduces aspects of cognitive empathy (Kamilar-Britt and Bedi 2015). In addition, evidence from multiple experimental paradigms (e.g. Bershad et al. 2016) supports the idea that MDMA’s subjective effects are mediated by supranormal central oxytocin levels (Francis et al. 2016). Since oxytocin is implicated in attachment behaviour, altruism and cooperation (Campbell 2010), its enhanced release could result in a strengthening of the therapeutic relationship and increase the willingness of patients to disclose private and painful memories during MDMA-assisted psychotherapy. In addition, MDMA might promote changes in dysfunctional *self*-referential attitudes, especially when used in conjunction with behavioural procedures that aim to modify these attitudes (Kamboj et al. 2015).

The goal of changing dysfunctional self-attitudes and beliefs (e.g. ‘I am worthless/unloveable’) reflects a general challenge in psychotherapy, namely, to create conditions that enable patients to respond to their perceived personal shortcomings in a less harsh and self-critical manner. In cognitive therapy, for example, this involves encouraging patients to generate compassionate responses to self-attacking thoughts (Kelly et al. 2009). These strategies have been refined into a system of psychological treatment—‘compassion focused therapy’—which integrates insights from Buddhist psychology with cognitive therapy (Gilbert 2010).

The inwardly directed affiliation promoted by techniques used in compassion-focused therapy, such as compassionate imagery, may also be generated or augmented by oxytocin release. In fact, intranasal oxytocin administration (Rockliff et al. 2011) and a common variant of the oxytocin receptor gene (Isgett et al. 2016) are associated with heightened self-compassion or prosocial and positive emotions after behavioural training intended to generate compassionate feelings. These observations contribute to our understanding of neurobiological mechanisms for building resilience and improving well-being, but also point to strategies for overcoming the pernicious negative emotional/cognitive states that characterise a variety of psychopathologies (Mennin and Fresco 2013). Such resilience can be inculcated through repeated and disciplined cognitive training regimes that enhance emotion regulation and down-regulate stress. An example of such training is mindfulness, although other approaches from Eastern meditative traditions—including compassion-oriented practices—have also been adopted in contemporary psychiatric and psychotherapy practice (D'Silva et al. 2012).

Despite the potential benefits of using compassion-oriented contemplative techniques as therapeutic strategies, individuals vary in their trait capacity for compassion and may find self-directed compassion-oriented strategies challenging, despite training. This has been conceptualised as a resistance to, or fear of, compassion and can represent a risk factor for psychopathology, or a barrier to successful treatment (Gilbert et al. 2012). Neuropsychopharmacological augmentation (e.g. through hyperstimulation of the oxytocinergic system) of psychosocial compassion-oriented strategies might mitigate against such barriers in those who are relatively insensitive to self-compassion-enhancing strategies. Such augmentation has been highlighted as an important area for psychiatric treatment development, especially for patients who are unresponsive to single-modality treatments, or for those who have special risk factors (Moss et al. 2016).

In a recent preliminary study, we found that recreational ecstasy use—which increases circulating oxytocin levels (Wolff et al. 2006)—was associated with an acute enhancement in self-compassion and reduction in self-criticism (Kamboj et al. 2015). The size of these effects was similar to those associated with use of a self-directed compassionate imagery (CI) task, and summation of the effects of MDMA and CI appeared to depend on participants’ adult attachment characteristics. However, since street ecstasy is often composed of various psychoactive substances other than MDMA, and the presence of MDMA was not assessed in that study (Kamboj et al. 2015), there are some justifiable concerns that psychoactive adulterants influenced those initial findings. More generally, data obtained under naturalistic conditions are also more likely to be influenced by ‘drug, set and setting’ (Zinberg 1986). This necessarily imposes some limits on the strength of conclusions that can be drawn from studies conducted in such settings. However, while obviously lacking the high levels of experimental control afforded by double-blind laboratory studies, ‘naturalistic studies’ (i.e. those conducted in ecological settings and/or with participants who are under the influence of a drug they have sourced themselves) are nonetheless potentially valuable in allowing efficient *preliminary* hypothesis testing on, as yet, poorly characterised drug effects. These can then pave the way for more tightly controlled studies if promising effects are observed. Previous naturalistic drug studies (Freeman et al. 2012; Morgan et al. 2010) have yielded novel findings that were subsequently independently replicated in double-blind laboratory experiments (de Sousa Fernandes Perna et al. 2016; Englund et al. 2013). Naturalistic studies of MDMA, in particular, may also have direct relevance to the reported self-experimental use of MDMA in those attempting to achieve personal growth through spiritual/contemplative practices (Watson and Beck 1991).

Here, we aimed to replicate previous findings on self-compassion and self-criticism following ecstasy use and/or CI (Kamboj et al. 2015), while also assessing the composition of participants’ self-administered ecstasy using high-precision spectroscopy. By only using data from participants whose ecstasy was confirmed to contain MDMA, and no other psychoactive compound, we aimed to demonstrate a clearer association between MDMA use and/or CI, and changes in self-attitudes (increased self-compassion and/or reduced self-criticism). On the basis of our previous findings (Kamboj et al. 2015), we hypothesised that recreational MDMA use and CI would be associated with similar increases in self-compassion and reductions in self-criticism. Furthermore, additive effects on these self-attitudes were hypothesised when MDMA and CI were combined. In addition, we examined the ostensibly separate and combined effects of MDMA and CI on general *inter*personal processing of criticism and compassion using a measure of emotional empathy that included critical and compassionate facial stimuli. Based on experimental studies of MDMA showing that it enhances emotional empathy in response to positive social stimuli (Kamilar-Britt and Bedi 2015), we predicted that it would enhance emotional arousal in response to compassionate facial expressions in the current study.

## Method

### Participants

Recreational ecstasy users were recruited by word-of-mouth and snowballing from the local community (note that we use the term ‘ecstasy’ when referring to the recreational drug when its composition is unknown and ‘MDMA’ in situations in which this constituent is known to be present). Interested participants were asked to contact the research team for information about the study. Those expressing an interest in participating were telephone-screened. Those disclosing a history of serious mental health problem or physical illness (i.e. requiring ongoing medical/psychiatric treatment), as well as women who were pregnant or likely to become pregnant or who were breast-feeding, were excluded.

Since our aim was to only include participants confirmed to have consumed MDMA, we over-recruited relative to our previous study (Kamboj et al. 2015), anticipating the exclusion of some volunteers. Twenty-five participants began and completed the study (14 men; 11 women). Data from 20 participants (12 men; 8 women) was retained as their ecstasy contained only MDMA (*n* = 18) or MDMA plus ≤ 33% glucose (*n* = 2). These participants had previous regular experience with MDMA (median length of experience, 4 years; median regularity of use, 1/month). Excluded participants’ ecstasy contained 50% MDMA plus 50% cocaine (*n* = 1), 67% MDMA plus 33% mephedrone/sucrose (*n* = 1), > 99% glucose plus undetermined impurities (*n* = 1), and < 50% MDMA plus > 50% sucrose plus solvent plus undetermined impurities (*n* = 2). The majority (*n* = 15) of participants used MDMA < 2 times/month (*n* = 5 used it ≥ 2 times/month). All participants also regularly (≥twice/month) consumed other drugs or alcohol: alcohol (*n* = 20), cocaine (*n* = 9), cannabis (*n* = 14), mephedrone (*n* = 6), amphetamine (*n* = 1), or other illicit drug (*n* = 3) and hence, are best characterised as polydrug users. Sixteen were smokers. Of the women participants, five used hormone-based contraception, and three were ‘regularly cycling’. The majority of participants (*n* = 17) did not practice any form of meditation. Participants received £30 for participating.

### Procedure

**Fig. 1:**
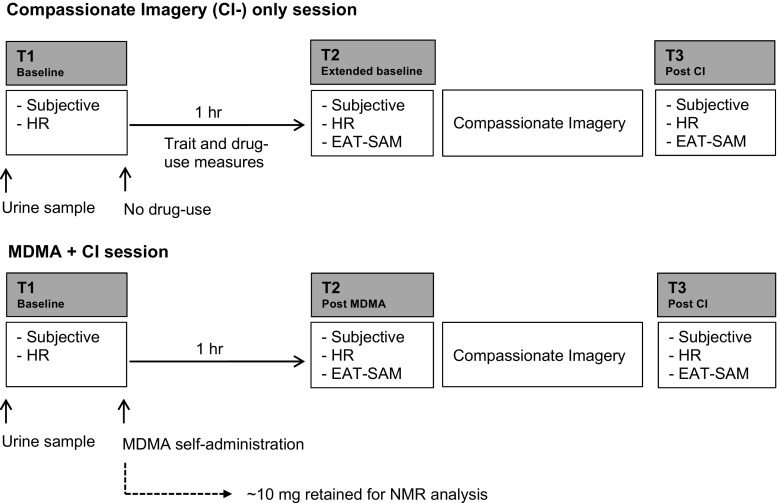
Protocol outline. All the participants completed both sessions. Subjective state measures (‘Subjective’ in the boxes under T1, T2 and T3) and heart rate (HR) were assessed at the three time points (T1, T2, T3) and emotional empathy (*EAT-SAM* empathy assessment task using the Self-Assessment Manikin) at T2 and T3. T1 corresponds to baseline, before MDMA use (on the MDMA + CI session). T2 was post-MDMA (on the MDMA + CI session) or served as a second, extended baseline measure (on the CI-only session). T3 was post-CI (both sessions). Procedure and assessment time points were the same on both sessions with the exception that no MDMA was self-administered and additional trait and drug-use history measures were completed (from T1 to T2) on the CI-only session

The procedure closely followed that used in Kamboj et al. (2015; see Fig. 1). A naturalistic (non-laboratory), within-subjects design was used, with all participants completing one session in which they took their usual dose of self-sourced ecstasy 1 h before a compassionate imagery (CI) task (the MDMA + CI session) and a second session (the CI-only session) in which they took no drug prior to CI. Testing sessions took place 6–14 days apart and were conducted in a quiet space in participants’ homes. Of the 20 participants whose ecstasy contained MDMA and no other drug (and whose data were therefore retained for analysis), 9 completed the MDMA + CI session first, followed by the CI-only session, meaning that session order was largely balanced across the participants.

The participants were asked to refrain from any drugs (except caffeine and nicotine) for 24 h prior to the testing session. A urine sample was provided at the start of each of the two sessions. ECG electrodes were attached and a period of stabilisation allowed. Affect state measures and a scenario-based measure of self-compassion/self-criticism (‘Subjective’ in Fig. 1) were taken at T1 (baseline), T2 (1 h after T1) and a third time point (T3; ~ 20 min after T2). After the subjective measures were completed at T2 and T3, an additional brief task was performed to assess emotional empathy (the Empathy Assessment Task using the Self-Assessment Manikin (EAT-SAM); see below). Since MDMA effects are close to peak levels after 1 h (Tancer and Johanson 2003), this was the fixed time interval used between T1 and T2 assessments, regardless of the route of administration. Between T1 and T2, the participants read or listened to music. A 1-h interval also separated T1 and T2 on the CI-only session. As such, T2 assessments served as an additional baseline in the CI-only session (see Fig. 1, top panel).

On the MDMA + CI session, participants provided the experimenter with the amount of drug they intended to consume to allow a small amount to be retained for testing, and the remainder was weighed prior to consumption. All MDMA was in powdered form and was consumed by insufflation (*n* = 3) or orally (*n* = 17), immediately after completing T1 assessments. The mean weight of consumed MDMA was 0.12 ± 0.03 g, with the following individually consumed amounts: 0.10 g (*n* = 5), 0.12 g (*n* = 8), 0.13 g (*n* = 5), 0.14 g (*n* = 1), and 0.25 g (*n* = 1). Note, the latter amount was an outlier (see below).

After repeating the state affect assessments at T2, the participants listened to guided CI instructions and then completed the state affect measures again at T3. The aim of the assessments at T1 and T2 was to assess state affect, self-compassion and self-criticism following MDMA use alone (i.e. prior to CI; MDMA + CI session), compared to the same T1–T2 period when MDMA was not consumed (CI-only session). At T3, we aimed to compare the combined effect of MDMA and CI (MDMI + CI session) with those of CI alone.

### Measures

#### Ecstasy-Related Mood States and Symptoms

A series of visual analogue scales assessed ecstasy-related mood and symptoms. Items included ‘energetic’, ‘anxiety’, ‘thirsty’, ‘muscle tension’, ‘hungry’, ‘jaw clenching’, ‘blurred vision’, ‘trust’, ‘empathy’, ‘sensitivity to colour’, ‘high’, ‘alert’, and ‘happy’. The anchors ‘not at all’ and ‘very’ (or ‘severe’ or ‘strong’) were used at the extremes of the scales (0 and 10).

#### State Self-Compassion and Self-Criticism

The Self-Compassion and Criticism Scale (SCCS; Falconer et al. 2015) is a scenario-based state measure of self-compassion and self-criticism. It consists of five scenarios designed to induce negative self-referential thinking. Participants imagined the scenarios and immediately rated their *current* reaction *towards themselves* in terms of self-reassurance, self-soothing, self-compassion (self-compassion subscale), self-contempt, self-criticism and self-harshness (self-criticism subscale) on a 1 (‘Not at all’) to 7 (‘Highly’) scale. This yields maximum subscale scores of 105 for self-criticism and 105 for self-compassion. The SCCS has been validated and demonstrates good psychometric properties (Cronbach’s alphas of 0.87 and 0.91 respectively for self-criticism and self-compassion subscales; Falconer et al. 2015) and is sensitive to change in response to compassion-oriented treatment strategies (Falconer et al. 2014; Kamboj et al. 2015).

#### Positive Affect

The Types of Positive Affect Scale (TPAS; Gilbert et al. 2008) assesses the extent to which people experience different types of positive affect using 18 adjective descriptors rated on a 5-point scale to indicate their current state (0 = ‘Not characteristic of me’, 4 = ‘Very characteristic of me’). Based on factor analysis, there are three subscales for the different types of positive affect: *active* (energetic, lively, adventurous, active, enthusiastic, dynamic, excited, eager; maximum score = 32), *relaxed* (peaceful, relaxed, calm, tranquil, laid back, serene; maximum = 24) and *content/warm* (safe, content, secure, warm; maximum = 16). The participants responded to items in relation to how they *currently felt*. Reported Cronbach’s alphas were 0.83 for active and relaxed affect and 0.73 for safe affect (Gilbert et al. 2008).

#### Depression

The 21-item Beck Depression Inventory-II (BDI-II; Beck et al. 1996) provided an assessment of severity of symptoms of depression. It is scored on a 0–3 scale, with higher values indicating higher levels of depressive symptoms (Cronbach’s *α* = 0.82; Richter et al. 1998).

#### Adult Attachment

The ‘close relationships’ version of the Revised Adult Attachment Scale was used (Collins 1996), which has three subscales (‘close’, ‘depend’ and ‘anxiety’; six items each). The ‘close’ subscale assesses the degree to which an individual is comfortable in close relationships and taps an aspect of attachment avoidance (e.g. ‘People often want me to be emotionally closer than I feel comfortable being’). The ‘depend’ subscale assesses a different aspect of attachment avoidance, namely, comfort/discomfort in depending on others and the extent to which participants believe others can be relied upon (e.g. ‘I know people will be there when I need them’). Higher scores are indicative of higher levels of comfort in depending on others (an analogue of secure attachment) and lower scores, greater discomfort (an analogue of insecure attachment). The ‘anxiety’ subscale taps a completely separate dimension of attachment that relates to concerns about being abandoned or rejected (e.g. ‘I want to get close to people, but I worry about being hurt’), with higher scores indicating higher levels of attachment anxiety. Items are rated on a 5-point Likert scale (0 = Not at all characteristic of me; 5 = Very characteristic of me). Cronbach’s alphas were reported as 0.77, 0.78 and 0.85, respectively for close, dependent and anxiety subscales (Collins 1996).

As previously described (Collins 1996), the ‘close’ and ‘depend’ subscales were strongly correlated in the current sample (*r* = 0.709, *p* < 0.001), whereas their correlation with attachment ‘anxiety’ was small (*r* = − 0.284 and − 0.273 respectively; *p* values ≥ 0.224). These subscale scores were entered as separate covariates to examine moderation of effects on self-compassion by individual differences in adult attachment.

#### Drug Use

Participants indicated whether they had consumed alcohol, tobacco, ecstasy, cannabis, cocaine, ketamine, hallucinogens, mephedrone or amphetamine in the past 24 h and previous 2 weeks. Urine samples were tested for the presence of these common recreational drugs (Alere, Abingdon, Oxfordshire, UK). Recent alcohol use was assessed using a breathalyser (Lion Instruments, UK) at the start of both sessions.

#### Physiological Assessment

An ambulatory ECG device (Firstbeat Bodyguard 2, Jyväskylä, Finland) was used to record heart rate (beats per minute derived from the recorded inter-beat intervals). One Ag/AgCl electrode was attached below the right clavicle and the other, below the left ribcage.

#### Empathy Assessment Task Using the Self-Assessment Manikin

We adapted this task from the one originally described by Ali et al. (2009); see also Seara-Cardoso et al. 2012). The Empathy Assessment Task using the Self-Assessment Manikin (EAT-SAM) was programmed in PsychoPy (Peirce 2007) and involved presenting and recording subjective arousal and valence responses evoked by facial affect stimuli comprising photographic images of basic (anger and happiness) and complex interpersonal (criticism and compassion) emotions (see Ali et al. 2009 for further details). Anger and happiness stimuli were drawn from the NimStim series (Tottenham et al. 2009) and consisted of nine different female actors. Since there are currently no similar standard stimuli of critical and compassionate faces, we used a new stimulus set consisting of nine facial expression of each, developed as part of a different project (Falconer et al., under review). The approach we used in generating these stimuli is outlined in detail in Tiddeman et al. (2001). Briefly, we first created neutral facial stimuli of nine different female identities, each of which was itself generated by averaging the photographed faces of three female actors using the image averaging software, PsychoMorph (Tiddeman et al. 2001). This software allows facial features from photographs to be registered with markers and then averaged into a composite of those features captured as a new image (and therefore new identity). The second stage involved morphing the facial features of the new (averaged) identities towards a composite prototype image of a face expressing either compassion or criticism (Perrett et al. 1994, 1998; Sprengelmeyer et al. 2009). These compassionate and critical facial stimulus composites were generated from 10 photographic images (out of an initial set of 128 different images) of different actors expressing compassion and criticism following an emotion-induction task relevant to generating these states. The selected photographs were those rated (by 70 raters) as most representative of compassionate and critical expressions (representative images available upon request from author CJF).

Immediately before the task, the participants were instructed on the meaning of the Self-Assessment Manikin (SAM) pictograms in terms of rating their current feelings on dimensions of arousal and valence, based on standard instructions for the SAM (Bradley and Lang 1994). The task was deployed on a PC when the participant indicated they understood the instructions. There were no practice trials. Individual facial expression images were preceded by a central fixation cross (1 s) and presented in randomised order on a 17-in. monitor until the participant responded to indicate their current arousal/valence in response to the facial expression. The next trial then began. Each scale was displayed below each affect image and consisted of nine radio buttons along with manikin-form anchors (Bradley and Lang 1994). Using a mouse, the participants selected one of the radio buttons for each scale to indicate their current emotional state *in response to* the images (1 = negative/low to 9 = positive/high) for each stimulus trial (each stimulus was presented once to assess valence, and once to assess arousal; 72 trials in total consisting of 9 different stimuli for each of four basic/complex expressions). The subjective arousal response to the affective displays of other people is considered an implicit measure of *emotional* empathy (Ali et al. 2009; Dziobek et al. 2008).

### Compassionate Imagery Task

Participants completed a guided CI exercise, as previously described (Kamboj et al. 2015). Briefly, the participants listened to standardised audio-recorded instructions presented through headphones describing the nature of compassion and the qualities of an ideal compassionate being. They then performed a guided ‘rhythmic breathing’ relaxation exercise and were guided on how to generate an image of an ideal compassionate being. The participants were told that this compassionate being could take any form they wished and that it would emanate compassion, directed at them.

### Analysis of Ecstasy Samples

Prior to consumption, a small amount of each participants’ ecstasy (~ 10 mg) was retained for subsequent analysis using a ^1^H nuclear magnetic resonance spectroscopy. A pure sample of MDMA was used as the reference material (≈ 99% pure by NMR). One- and two-dimensional NMR data were collected on a Bruker (500-MHz NMR) spectrometer. Mass spectra were recorded on a Bruker Daltonics ‘micrOTOF’ electrospray ionization mass spectrometer (ESI-MS). Each sample was weighed, labelled and dissolved in deuterated methanol (0.05% TMS) before analysis. Samples that were found to be impure by ^1^H NMR, or that lacked MDMA, were further analysed using ^13^C, DEPT, COSY, HMBC and HSQC spectroscopy in order to determine the identity of the other constituents.

### Data Analyses

The Statistical Package for Social Sciences (SPSS, version 24, IBM) was used to perform all statistical analyses. Data was examined graphically and statistically for normality and outliers. Shapiro-Wilk tests were non-significant (all *p* values > 0.1) and no studentised residuals exceeded ± 3 on the outcomes. Parametric statistical analyses were therefore applied. Two-way Time × Session repeated measures ANOVAs, with Session (CI-only or MDMA + CI) and Time (T1, T2 and T3) as within-subject factors, were used to analyse positive/negative mood states and state self-compassion and self-criticism. Three-way (Time × Session × Emotion) ANOVAs were used to analyse the EAT-SAM arousal responses. For the latter, the Time factor had only two levels (T2, T3) and separate analyses were conducted for basic (angry; happy) and complex emotions (critical; compassionate) because these pairs of stimuli were drawn from different sources. As such, the Emotion factor also had two levels (angry and happy *or* critical and compassionate). Additional ANCOVAs were used to assess moderation by attachment security of the effects of MDMA and CI on self-compassion. Specifically, indices of dispositional avoidant attachment (closeness and dependence scores on the AAS) were entered as covariates.

Planned comparisons were used to follow up the main analyses on self-compassion and self-criticism. All other post-hoc analyses of significant ANOVA effects were Bonferroni-corrected. Pearson’s correlations were used to explore association between variables. All reported statistics are two-tailed and values are presented as means ± standard deviations unless otherwise indicated (in figures). As reflected in the reported dfs, the analyses were based on complete data, except for heart rate, which, due to technical difficulties, was missing at one or more time points for two participants. ANOVA effect sizes (*η*_p_^2^) were calculated in SPSS, and individual effect sizes (Cohen’s *d*) included an adjustment for the correlation between paired observations where appropriate (Lenhard and Lenhard 2015).

Key analyses were repeated excluding the participant with the outlying MDMA dose (0.25 g). Similarly, re-analyses was conducted by excluding the three participants who insufflated their MDMA. The patterns of means, effect sizes and statistical significance (i.e. < 0.05) were retained despite data removal, and as such, all the 20 cases were retained in the analyses.

## Results

The mean age of the participants (12 men, 8 women) was 28.45 ± 6.16 years. They had 15.05 ± 2.35 years of education and BDI scores of 9.50 ± 6.53. All the participants provided 0.00% readings on the alcometer on both sessions. All participants reported abstinence from drugs (including alcohol) in the 24 h prior to both testing sessions, but all also reported use of some illicit substance in the 2 weeks preceding one or both sessions. Urine tests on the CI-only session were either negative for all drugs (*n* = 7), positive for THC (*n* = 6), cocaine (*n* = 5), MDMA (*n* = 1), opioids (*n* = 1) or benzodiazepines (*n* = 3). On the MDMA + CI session, urine tests were negative for all drugs (*n* = 10) or positive for amphetamine (*n* = 2), THC (*n* = 6; *n* = 5 of whom tested positive for THC on the CI-only session), opioids (*n* = 1; who also tested positive on CI-only session) and benzodiazepines (*n* = 3; *n* = 1 of whom tested positive on CI-only session).

### Subjective Effects of MDMA: Visual Analogue Scales

There were significant Time × Session interactions for thirst, energetic, muscle tension, jaw clenching, blurred vision, sensitivity to colour, hunger, high and alertness. These interactions reflected a general pattern of increases (or a decrease, in the case of hunger) from T1 to T2/T3, only on the MDMA + CI session. Main effects of Session on anxiety, trust and happy reflected higher levels of these states on the MDMA + CI session and main effects of Time on trust, empathy and happy reflected lower levels of these states at T1. The pattern of effects is not consistent with increasing MDMA effects between T2 and T3 on the MDMA + CI session. In fact, when the above analyses were repeated with only two levels of the Time factor (T2, T3), three of the ANOVAs showed a Time × Session interaction, with muscle tension (*p* = 0.011) and jaw clenching (*p* = 0.002), evidencing an increase between T2 and T3 on the MDMA + CI session, whereas ‘high’ showed a *decrease* on the MDMA + CI session (*p* = 0.004). The remaining 2 × 2 interactions were non-significant (*p* values > 0.1). As such, it is unlikely that differential effects (reported below) across sessions and between T2 and T3 (the period when the CI task was performed) can simply be attributed to continued increases in response to MDMA between T2 and T3, rather than to the additional effects of CI.

### Physiological Effects

An expected Session × Time interaction was found (*F*(1.4, 23.5) = 9.034, *p* = 0.003, *ɳ*_p_^2^ = 0.347) for heart rate. This reflected a sustained increase in heart rate in the MDMA + CI session. Specifically, compared to T1, heart rate was 14.15 ± 18.93 beats/min higher at T2 (*t*(17) = 3.171, *p* = 0.006) and 14.63 ± 19.39 beats/min higher at T3 (*t*(17) = 3.200, *p* = 0.005) on the MDMA + CI session relative to the CI-only session.

### Effects on Self-Criticism and Self-Compassion

**Fig. 2:**
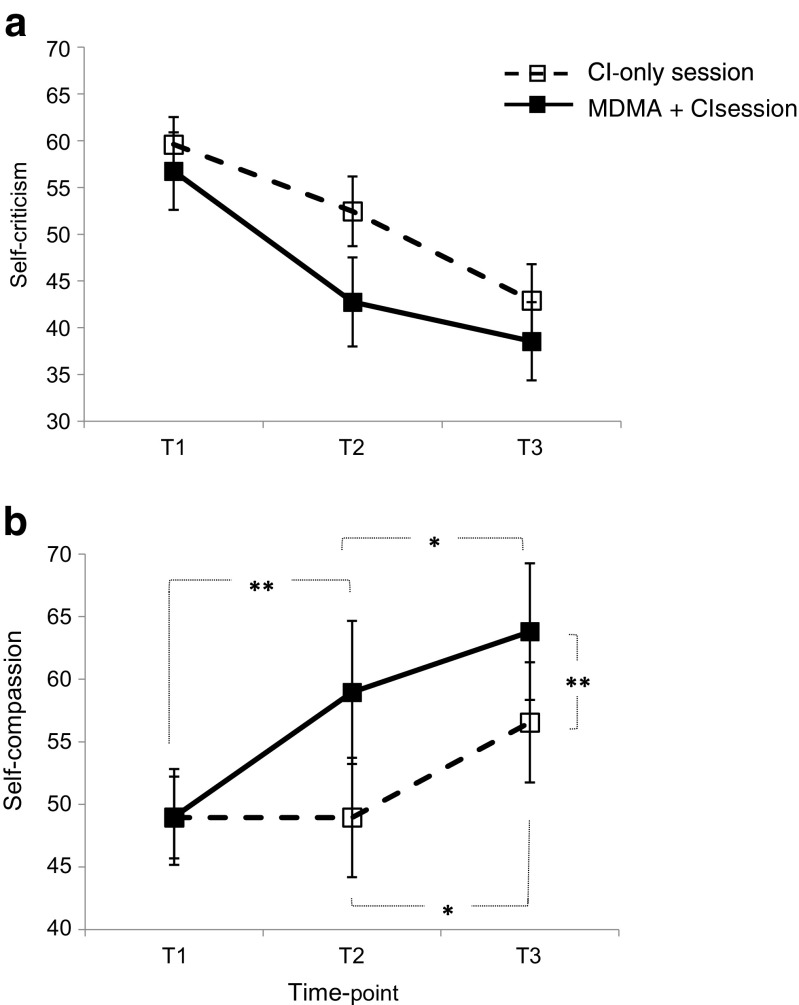
**a** Mean (± SEM) SCCS self-criticism scores at three time points: T1 (baseline), T2 (the extended baseline/post-MDMA time point) and T3 (post-CI on both sessions). **b** Mean (± SEM) SCCS self-compassion scores recorded at the same time points (T1–T3). The dashed line represents results from the CI-only session, and the solid line, the MDMA + CI session. One asterisk indicates *p* < 0.05; two asterisks indicate *p* < 0.01 in post-hoc tests

On the state self-criticism subscale of the SCCS, there were main effects of Time (*F*(2,38) = 26.745, *p* < 0.001, *ɳ*_p_^2^ = 0.585) and Session (*F*(1,19) = 6.881, *p* = 0.017, *ɳ*_p_^2^ = 0.266), but the Time × Session interaction was not significant (*F*(2,38) = 2.584, *p* = 0.089, *ɳ*_p_^2^ = 0.120; Fig. 2a). The state compassion subscale of the SCCS also showed main effects of Time (*F*(1.5,28.1) = 10.285, *p* = 0.001, *ɳ*_p_^2^ = 0.351) and Session (*F*(1,19) = 6.592, *p* = 0.019, *ɳ*_p_^2^ = 0.258). However, these were subsumed by a Time × Session interaction (*F*(2,38) = 5.794, *p* = 0.006; *ɳ*_p_^2^ = 0.351; Fig. 2b). Follow-up analyses showed that on the CI-only session, there was no change in self-compassion during the extended baseline (T1–T2; *p* = 1.00; Fig. 2b), but there was an expected increase between T2 and T3, after CI (*p* = 0.023, *d* = 0.355). On the MDMA + CI session, there was a significant increase in self-compassion between T1 and T2 (*p* = 0.008, *d* = 0.396), ostensibly reflecting the effect of MDMA alone. There was an additional, albeit small, increase between T2 and T3 (*p* = 0.015, *d* = 0.191) on the MDMA + CI session, putatively reflecting the combined effects of MDMA and CI on self-compassion. SCCS self-compassion scores were not different at T1 on the two sessions (Fig. 2b) (*p* > 0.1, *d* = 0.03), but diverged significantly at T3, with higher state self-compassion on the MDMA + CI session (*p* = 0.003, *d* = 0.301). Overall, this pattern of effects is consistent with enhancement of the effects of CI on self-compassion in the presence of MDMA.

To determine whether these subjective effects of CI ± MDMA on self-compassion were specific, or reflected a more general pattern of effects on positive affective states, we examined the effects of CI ± MDMA on the TPAS active, relaxed and content/warm subscales (Table 1). However, we found no Session × Time interactions on any of the TPAS subscales (*F* values ≤ 2.1, *p* values > 0.1). Even when considering only the first two time points (i.e. comparing the effects of MDMA self-administration with the extended baseline on the CI session), only the active subscale showed signs of a potential interaction (*F*(1,19) = 3.252, *p* = 0.087), with higher levels of active positive affect post-MDMA (at T2). The other two subscales showed no interaction (*F* values < 1).

**Table 1:** Three types of positive affect (TPAS; active, relaxed and warm/content) at three time points (T1, T2 and T3) by session (mean ± SD)

	CI session			MDMA + CI session			*F* value		
	T1	T2	T3	T1	T2	T3	Session	Time	Session × Time
TPAS—active	16.10 (6.04)	15.40 (5.85)	11.80 (5.31)	18.80 (5.07)	22.05 (5.81)	17.55 (6.07)	27.808***	12.00***	2.056
TPAS—relax	15.15 (4.61)	16.05 (4.14)	18.50 (5.10)	14.75 (5.31)	15.40 (4.60)	20.35 (4.18)	0.110	16.056***	1.770
TPAS—warm	11.10 (2.65)	12.05 (2.24)	12.80 (2.55)	12.35 (2.28)	12.80 (2.91)	14.15 (1.90)	5.652*	8.591**	0.450

*p < 0.05

**p ≤ 0.01

***p ≤ 0.001

### Moderating Role of Attachment on Self-Compassion

We did not observe a moderating role for the Closeness aspect of attachment avoidance in the Time × Session interaction on self-compassion (*F* < 1; cf. Kamboj et al. 2015). The Time × Session × Attachment analysis with AAS—dependence scores as a covariate did not reach statistical significance (*F*(2,36) = 3.039, *p* = 0.06, *ɳ*_p_^2^ = 0.144), although descriptively/graphically, it appeared that those with higher AAS—dependence scores showed larger increases in self-compassion in response to CI or MDMA whereas low scorers were relatively insensitive to both CI and MDMA.

### Emotional Empathy

**Fig. 3:**
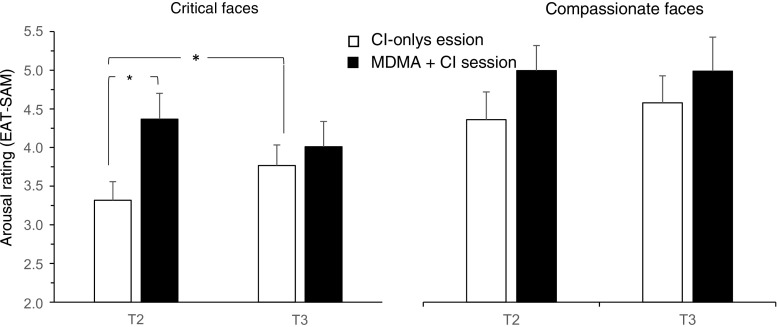
Mean (± SEM) arousal ratings (indexing emotional empathy) in response to critical and compassionate faces on the empathy assessment task using the Self-Assessment Manikin (EAT-SAM). Two asterisks indicate *p* < 0.01 for MDMA versus drug-free performance on critical faces at T2; one asterisk indicates *p* < 0.05 for the T2 versus T3 effect of CI only on critical faces

On the arousal measure of the EAT-SAM, there were no effects of Session or Time (*F* values ≤ 2.753, *p* values > 0.1) for basic emotions, although there was a main effect of Emotion (anger > happiness; *F*(1,19) = 11.756, *p* = 0.003). Similarly for valence, there was only a main effect of Emotion (happiness > anger; *F*(1,19) = 26.893, *p* < 0.001), but no effects of Session or Time (*F* values ≤ 3.047, *p* values ≥ 0.097). For the complex emotion duo (critical and compassionate faces), there was a significant Time × Session × Emotion interaction on the arousal measure of the EAT-SAM (*F*(1,19) = 5.155, *p* = 0.035, *η*_p_^*2*^ = 0.213; Fig. 3), which was investigated further with separate Time × Session ANOVAs for compassionate and critical facial stimuli.

Critical faces showed a significant Time × Session interaction (*F*(1, 19) = 16.429, *p* = 0.001 *η*_p_^2^ = 0.464). Pairwise comparisons across levels of Session and Time showed that at T2 (which assesses the effect of MDMA relative to extended baseline, prior to CI), arousal in response to critical faces was higher on the MDMA + CI session (*p* < 0.001, *d* = 0.768). In addition, there was an increase in arousal in response to critical faces between T2 and T3 on the CI-only session (*p* = 0.019, *d* = 0.391), reflecting the effects of CI alone. This suggests an increase in arousal in response to critical faces after either intervention separately, although the effect of CI alone was substantially smaller than that of MDMA.

There was no evidence of an additive response to MDMA and CI on EAT-SAM arousal (between T2 and T3 on the MDMA + CI session). In addition, there was only a main effect of Emotion for the complex emotions on the valence measure of the EAT-SAM (compassionate > critical; *F*(1,19) = 28.466, *p* < 0.001; other effects: *F* values ≤ 1). Possible associations were examined between EAT-SAM arousal and valence ratings to critical faces at T2 and T3, with Self-Compassion and Self-Criticism scores at the same time points but no significant correlations emerged.

## Discussion

In this report, we extend previous findings on the effects of recreational ecstasy and compassionate imagery (CI) on self-attitudes. The broad qualitative pattern of individual effects of MDMA and CI reported previously (Kamboj et al. 2015) were also found here, although the statistical pattern of interactions diverged. In particular, in our previous study, we found clearer evidence of additive effects of MDMA and CI on self-criticism (here, the Time × Session interaction was suggestive but not statistically significant). Critically, in the current study, we only used data from participants whose ecstasy was confirmed to contain only MDMA (plus ≤ 33% glucose in two samples). The broadly similar pattern of effects across both studies supports the validity of those previous findings, although the confirmed sole presence of MDMA in participants’ ecstasy in this study lends it more weight.

The primary findings in the current study were that MDMA and CI appeared to produce similar (small to medium) increases in self-compassion when administered separately. Note however that these effects were not based on placebo-controlled comparisons and must therefore be considered provisional. When the CI instructions occurred in the presence of MDMA, there was a small but significant further increase in self-compassion, suggesting that the effects of CI can be potentiated in the presence of MDMA. In addition, emotional empathy in response to critical faces appeared to increase following CI (the small within-session increase in EAT-SAM arousal between T2 and T3 on the CI-only session) and following MDMA (the medium to large between-session difference in EAT-SAM arousal at T2).

The effects on self-compassion were found in the absence of interactions on three relevant and psychometrically distinct types of positive affect (relaxation, contentment/warmth and activation). It has been suggested that arousal ratings in tasks such as the EAT-SAM represent an *implicit* index of emotional empathy (Dziobek et al. 2008). As such, our main findings are not entirely consistent with a simple generalised enhancement of positive affect or demand characteristics. In contrast to these effects on self-compassion, the effects of CI ± MDMA were less clear-cut for self-criticism. Examination of Figure 2a shows that there was an unexpected reduction in self-criticism in the absence of MDMA and before CI (i.e. between T1 and T2), which may explain the lack of a Time × Session interaction (*p* = 0.089; cf. Kamboj et al. 2015).

Although previous studies have also examined the effects of MDMA on emotional empathy, these have tended to use situational rather than facial stimuli (reviewed by Kamilar-Britt and Bedi 2015) and have not specifically examined compassion and criticism. Unlike these previous studies, we did not find a generalised increase in emotional empathy or the more specific hypothesised increase in response to compassionate expressions in the presence of MDMA (cf Kamilar-Britt and Bedi 2015). Instead, while there was no effect on EAT-SAM arousal to basic emotion or compassionate facial expressions in response to CI ± MDMA, critical faces elicited enhanced arousal following MDMA at T2 relative to the extended baseline time point (T2) on the CI-only session. In addition, CI also appeared to increase arousal to critical faces (from T2 to T3 on the CI-only session). The specific effects of MDMA on critical (i.e. negative) expressions diverged from previous studies, which have not tended to show such enhancement of emotional empathy in response to negatively valenced stimuli. This divergence may relate to our use of facial (rather than situational) affective stimuli generally or critical facial expressions in particular, which have not been used in previous studies.

The apparently similar effects of CI and MDMA (on state self-compassion rather than positive affect more generally, and on arousal to critical facial stimuli, rather than other emotion expressions) might suggest a common biopsychological mechanism through which some behavioural and pharmacological strategies increase aspects of self-affiliation and emotional empathy. However, given the distinct effects of MDMA on the sympathetic system (Clark et al. 2015) and compassion-oriented practices on the parasympathetic system (Stellar et al. 2015), it seems unlikely that the qualitatively similar effects of CI and MDMA observed here are due to common effects on the autonomic nervous system. Alternatively, given its ostensible role in (self-) affiliation (Rockliff et al. 2011) and its hyperstimulation by MDMA (Bershad et al. 2016), the oxytocinergic system seems to be a mechanistically plausible substrate for the common effects observed here, at least for self-compassion.

Our current and previous (Kamboj et al. 2015) findings complement the existing biobehavioural methods used in the neuroscientific study of contemplative practices (Ricard et al. 2014). MDMA’s effects are particularly relevant to contemplative neuroscience in terms of mimicking or augmenting the effects of compassion-oriented practices (e.g. loving kindness meditation or compassion-focused therapy) through a biologically plausible (oxytocinergic) pathway. Whether the observations reported here have therapeutic relevance, however, remains an open question and would need to be the subject of separate careful study. A primary concern in such studies would be to determine if lasting beneficial effects (on self-attitudes) would be observed after a small number of doses of MDMA in combination with a compassion-oriented psychosocial strategy (such as CI). If so, this would be in line with the existing model of MDMA-assisted psychotherapy for posttraumatic stress disorder (Mithoefer et al. 2013). Moreover, it may be that such combination treatments would be applicable in those with specific vulnerabilities (e.g. insecure attachment or fear of/resistance to compassion) who might otherwise fail to benefit from compassion-oriented psychosocial treatments (e.g. compassion-focused therapy).

### Limitations

While our assessment of MDMA composition represents a methodological improvement to our previous study design, naturalistic studies have some clear limitations and can only be considered to be a starting point, paving the way for investigating the relevant phenomena in tightly controlled experimental contexts. The setting within which this study was conducted and critically, the participant group in whom it was conducted are important considerations. As a result, we cannot know, for example, the degree to which the findings would generalise to MDMA-naive participants or to those with whom compassion-oriented strategies are typically used in the context of psychopathology (Gilbert 2010). Moreover, while highly controlled laboratory settings are clearly the preferred context within which to test drug effects, when subjective (rather than implicit behavioural) effects are the primary outcome, concerns about expectancy are not eliminated. In particular, it is not possible to retain blinding of participants once they become aware of the drug’s subjective effects, especially if they have previous experience with MDMA (which is commonly the case in related studies; Kamilar-Britt and Bedi 2015).

In addition, since participants chose the amount of MDMA they consumed (presumably based in their previous experience with the drug, and expectations about its likely effects on them), this inevitably introduced some variability in the effects of the drug. On the other hand, except for one outlier, the absolute weight of participants’ MDMA fell within a rather narrow band (0.1–0.14 g). However, another potential limitation was that, while the participants indicated verbally that they had not consumed any drugs in the 24 h prior to testing, a sizeable proportion provided positive urine screens for illicit drugs. However, the slow clearance of some drugs means that their presence in urine does not necessarily imply non-compliance with abstinence instructions in the preceding 24 h. THC and benzodiazepines in particular have prolonged elimination kinetics (Moeller et al. 2008). However, apart from alcohol (which all the participants provided negative readings for), a more fine-grained quantitative analysis of recent drug use was not employed here. The absence of confirmatory chromatographic/spectrometric analysis of positive drug screens leaves some uncertainty about the role other drugs might have played in the effects reported here.

We aimed to use the most efficient design possible to preliminarily test our hypotheses. As such, additional control conditions (e.g. an MDMA-only condition, and a control behavioural strategy for CI) were not employed. Future tests of these ideas in laboratory-controlled experiments would need to carefully consider the issue of the number of control conditions and experimental sessions, as it is unclear whether the outcome measures used here would retain sensitivity to change within a more intensive testing schedule. Alternatively, less efficient between-subjects designs would be required.

Overall, our findings suggest that further research on adaptive modification of intrapersonal attitudes following MDMA (and its combined use with compassion-oriented behavioural strategies) is warranted. Future related studies should employ randomised, double-blind controlled designs. Rigorous tests of the suggestive findings reported here could eventually inform the way in which MDMA (or similar ‘entactogens’ that might be developed in the future) is used in combination with psychotherapeutic strategies. Clearly, future work in this area requires continued efforts by researchers and advocates of ‘psychedelic’ research to persuade regulators and funders that this is a legitimate area of research, with potentially important implications for the treatment of psychological disorders (Nutt et al. 2013).
